# 2021 U.S. Virgin Islands Zika health brigade: Providing recommended pediatric health screenings for children born to mothers with laboratory evidence of possible Zika virus infection during pregnancy

**DOI:** 10.1002/bdr2.2143

**Published:** 2022-12-27

**Authors:** Leah H. de Wilde, Cosme Jeremy Harrison, Binta E. Ceesay, Charmaine S. Mayers, Janney Ferrol-Hawley, Jacqueline Canton, Shana Godfred-Cato, Megan R. Reynolds, Lessely Brown-Shuler, Sukhdeep Randhawa, Dan Schoelles, Braeanna Hillman, Maria Paz Carlos, Tracey Ambrose, Derek Bitner, Sandra Holgado, Cristie Jones, Daniel J. Lattin, Sarah B. Mulkey, Angeline Nguyen, Mary Payne, S. Grace Prakalapakorn, Ann Shue, Esther M. Ellis

**Affiliations:** 1 U.S. Virgin Islands Department of Health, Christiansted and Charlotte Amalie, Virgin Islands, USA; 2 Chickasaw Nation Industries, Norman, Oklahoma, USA; 3 Division of Birth Defects and Infant Disorders, National Center on Birth Defects and Developmental Disabilities, Centers for Disease Control and Prevention, Atlanta, Georgia, USA; 4 Deloitte Consulting LLP, New York, New York, USA; 5 Maternal Child Health Bureau (MCHB), Health Resources and Services Administration (HRSA), U.S. Department of Health and Human Services (HHS), Washington, D.C., USA; 6 Hearing and Speech Center, Children's National Hospital, Washington, D.C., USA; 7 Wolfe Eye Clinic, West Des Moines, Iowa, USA; 8 Duke Eye Center, Durham, North Carolina, USA; 9 Austin Regional Clinic, Austin, Texas, USA; 10 Nemours Children's Health, Jacksonville, Florida, USA; 11 Prenatal Pediatrics Institute, Children's National Hospital, Washington, D.C., USA; 12 Department of Neurology, The George Washington University School of Medicine and Health Sciences, Washington, D.C., USA; 13 Department of Pediatrics, The George Washington School of Medicine and Health Sciences, Washington, D.C., USA; 14 Pediatric Ophthalmology and Adult Strabismus, Children's Hospital Los Angeles, Los Angeles, California, USA; 15 Keck School of Medicine, University of Southern California, Los Angeles, California, USA; 16 Marshall University School of Medicine, Huntington, West Virginia, USA; 17 Department of Ophthalmology, Duke University, Durham, North Carolina, USA; 18 Department of Pediatrics, Duke University, Durham, North Carolina, USA; 19 Duke Global Health Institute, Duke University, Durham, North Carolina, USA; 20 Byers Eye Institute, Department of Ophthalmology, Stanford University, Palo Alto, California, USA; 21 Stanford Children's Health, Palo Alto, California, USA

**Keywords:** congential Zika virus, health brigade, health screening, pediatric, pediatric screening, specialty care, Zika virus infection

## Abstract

**Background:**

The United States Virgin Islands (USVI) Department of Health (DOH) conducted a second Zika health brigade (ZHB) in 2021 to provide recommended Zika-related pediatric health screenings, including vision, hearing, neurologic, and developmental screenings, for children in the USVI. This was replicated after the success of the first ZHB in 2018, which provided recommended Zika-related pediatric health screenings to 88 infants and children exposed to Zika virus (ZIKV) during pregnancy.

**Methods:**

Ten specialty pediatric care providers were recruited and traveled to the USVI to conduct the screenings. USVI DOH scheduled appointments for children included in CDC's U.S. Zika Pregnancy and Infant Registry (USZPIR). During the ZHB, participants were examined by pediatric ophthalmologists, pediatric audiologists, and pediatric neurologists. We report the percentage of participants who were referred for additional follow-up care or given follow-up recommendations in the 2021 ZHB and compare these referrals and recommendations to those given in the 2018 ZHB.

**Results:**

Thirty-three children born to mothers with laboratory evidence of ZIKV infection during pregnancy completed screenings at the 2021 ZHB, of which 15 (45%) children were referred for additional follow-up care. Ophthalmological screenings resulted in the highest number of new referrals for a specialty provider among ZHB participants, with 6 (18%) children receiving referrals for that specialty. Speech therapy was the most common therapy referral, with 10 (30%) children referred, of which 9 (90%) were among those who attended the 2018 ZHB.

**Conclusions:**

Thirty-three children in a jurisdiction with reduced access to healthcare specialists received recommended Zika-related pediatric health screenings at the ZHB. New and continuing medical and developmental concerns were identified and appropriate referrals for follow-up care and services were provided. The ZHB model was successful in creating connections to health services not previously received by the participants.

## 1 – BACKGROUND

From July 26 to August 6, 2021, the United States Virgin Islands (USVI) Department of Health (DOH) hosted the second pediatric Zika health brigade (ZHB) on the islands of St. Croix and St. Thomas to provide recommended Zika-related health screenings for children exposed to Zika virus (ZIKV) during pregnancy (Adebanjo et al., 2017). The USVI DOH collaborated with the Centers for Disease Control and Prevention (CDC) in engaging the Centers for Medicare and Medicaid Services and American Academy of Pediatrics (AAP) to replicate the services provided during the 2018 ZHB (Hillman et al., 2019) (Godfred-Cato et al., 2020). While services were extended to all infants and children aged 0–5 years in the territory during the ZHB, for the purpose of this report, we only provide results for children born to mothers with laboratory evidence of possible ZIKV infection during pregnancy. The second ZHB, initially scheduled for March 2020, was postponed due to the COVID-19 pandemic. The ZHB was ultimately rescheduled for July 2021 and aimed to provide recommended age-appropriate Zika-related pediatric health and developmental screenings for children born to mothers with laboratory evidence of possible ZIKV infection during pregnancy. We report the percentage of participants who were referred for additional follow-up care or given follow-up recommendations in the 2021 ZHB and compare these referrals and recommendations to those given in the 2018 ZHB to understand how Zika continues to affect children in the USVI.

From December 1, 2015 to March 31, 2018, USVI DOH reported cases to CDC's U.S. Zika Pregnancy and Infant Registry (USZPIR), which monitors health outcomes among infants and children born to women with laboratory evidence of possible ZIKV infection during pregnancy (Shapiro-Mendoza et al., 2017). In the USVI, 287 women with ZIKV infection during pregnancy were reported to and monitored for the USZPIR, with 250 known live births, which includes one infant death. The USVI collects health information about children born to these mothers up to age 5 years, with 125 children still actively monitored. All infants born to mothers with possible ZIKV exposure during pregnancy were recommended to receive a standard evaluation at birth and at each subsequent well-child visit, including a comprehensive physical examination, laboratory testing for ZIKV, head ultrasound by age 1 month, age-appropriate vision screening, newborn hearing screen, preferably using auditory brainstem response (ABR) by 1 month of age, and developmental monitoring and screening using validated tools (Adebanjo et al., 2017).

Access to pediatric specialty screenings for families in the USVI continues to be an ongoing challenge. Barriers to access include lack of specialty pediatric providers in the territory, cost of screenings, the complexity of continuity of care between on-island and off-island visits, as well as the coronavirus disease 2019 (COVID-19) pandemic decreasing access to care. Pediatric specialty care is often only available through intermittent traveling providers coordinated by USVI DOH's Maternal and Child Health offices, travel to off-island medical facilities, or through adult providers seeing children. Currently, there is only one pediatric neurologist practicing in the USVI through quarterly travel to the territory, and no pediatric audiologists, ophthalmologists, or developmental pediatricians (Hillman et al., 2019) (Godfred-Cato et al., 2020). The health brigade model addresses these challenges by providing families with the opportunity to receive all four specialty screenings and recommendations for follow-up referrals at one time, free of charge, and without off-island travel.

## 2 ∣ METHODS

The USVI DOH conducted outreach to cases monitored through the USZPIR to participate in the ZHB, including those considered lost to follow-up. Responses were limited due to inaccurate contact information. Several families that were reachable expressed time constraints, not being on island at the time of the brigade, or disinterest. Additional outreach efforts were made through community and provider engagement by DOH officials, health brigade organizers, local pediatric providers, and USVI collaborating agencies including the Infants and Toddlers Program; Women, Infants and Children; Early Head Start; Department of Human Services; Department of Special Education; and Island Therapy Solutions. Outreach included contacting families via phone, flier distribution, notification from pediatric health care providers during clinic visits, and public service announcements through radio and social media to increase awareness of the importance of developmental and physical screenings.

Working with the AAP, CDC recruited 10 pediatric specialty providers for the 2021 ZHB, including five pediatric ophthalmologists, one orthoptist, two pediatric audiologists, and two pediatric neurologists. Each ZHB participant rotated through four specialty screenings during one clinic appointment, receiving age-appropriate vision, hearing, neurologic, and developmental screenings. Pediatric ophthalmologists and an orthoptist performed all ophthalmologic examinations, consisting of an evaluation of visual function, visual development, ocular motility and alignment, stereopsis, intraocular pressure, anterior and posterior segment examinations, and cycloplegic refraction. Pediatric audiologists performed testing specific to each patient's developmental age and tolerance, including Automated Auditory Brainstem Response (AABR), Tympanometry, Distortion Product Otoacoustic Emissions (DPOAEs), Visual Reinforcement Audiometry (VRA), ipsilateral Acoustic Reflex, Conditioned Play Audiometry, and Conventional Audiometry. Pediatric neurologists performed the Ages and Stages Questionnaire, third edition (ASQ-3) developmental screening; the Ages and Stages Questionnaire: Social Emotional, second Edition; the Modified Checklist for Autism in Toddlers; and a full neurological examination. These evaluations are those provided during the 2018 ZHB but adjusted to align with the increased ages of the children.

Preexisting COVID-19 protocols employed by USVI DOH were followed to ensure the safety of staff and participants, such as temperature checks, symptomology and exposure checklists, donning personal protective equipment, social distancing, and routine sanitization. During the exit process of the ZHB, a summary of clinical findings was given to each family, which included recommended referrals for future follow-up with specialists to share with their primary pediatric care providers. Recommendations and referrals for early intervention and therapies were shared with the necessary USVI DOH and ZHB partners to be reviewed for enrollment. We report the percentage of participants who were referred for additional follow-up care or given follow-up recommendations in the 2021 ZHB and compare these referrals and recommendations to those given in the 2018 ZHB to understand how Zika continues to affect children in the USVI.

## 3 ∣ RESULTS

Of the children tracked for the USZPIR in USVI, 43 scheduled an appointment and 33 attended and completed the screenings at the 2021 ZHB (Table 1); the 2018 ZHB included 88 children tracked by the USZPIR (Table 2). Of the participants from the 2021 ZHB, 24 (73%) children had also participated in the 2018 ZHB (Table 1). Of all 33 of the 2021 ZHB participants, 15 (45%) were referred for additional follow-up care (Table 2). Referral for follow-up care was defined as instructions for a follow-up appointment with a specialty healthcare provider or recommendation to enroll in therapy to address a developmental or physical concern noted during the health brigade. Ophthalmological screenings resulted in the highest number of new referrals and recommendations for a specialty provider from all 33 participants, with 6 (18%) children receiving referrals. In terms of therapies, referral to speech therapy was most frequent with 10 (30%) children referred. Communication was the most affected domain on the ASQ-3, with 11 (33%) children receiving a borderline or below-cut-off score and needing further assessment (Table 2).

Of the 24 participants who returned from the 2018 ZHB, 12 (50%) received referrals and were recommended for follow-up. Of the 12 participants from this group that received referrals and recommended follow-up care, 9 (75%) were referred for speech therapy, making speech therapy the most common therapy referral among returning participants from the 2018 ZHB. Of note, these nine speech therapy referrals accounted for 90% (9/10) of the total speech therapy referrals during the 2021 ZHB. Among the same group of returning participants from the 2018 ZHB, 3 (13%) participants were recommended to continue receiving previously prescribed therapies. These previously prescribed therapies were self-reported from participant's guardians to the specialty healthcare providers during the screenings. Among the 12 participants who returned from the 2018 ZHB that did not receive referrals or recommended follow-up at the 2021 ZHB, 9 (75%) had received referrals or recommended follow-up at the 2018 ZHB. For the nine participants who attended the 2021 ZHB but did not attend the 2018 ZHB, 3 (33%) children received new referrals and recommendations. One participant received referrals for physical therapy, speech therapy, an autism evaluation, and a follow-up referral with a developmental pediatrician. One participant received a follow-up referral with a pediatric neurologist, and a referral for neuroimaging. The third participant received a follow-up referral with an ophthalmologist. None of these children were receiving therapy at the time of the 2021 ZHB.

We compared specialty care referrals made during the 2018 ZHB to specialty care referrals made during the 2021 ZHB. In descending order, top referrals from the 2018 ZHB were made for audiology, the Infants and Toddlers Program, and physical therapy. From the 2021 ZHB, top referrals were made for speech therapy, ophthalmology, and neurology. This cohort aged out of the Infants and Toddlers Program, but one child was referred to Head Start (Table 2).

## 4 ∣ DISCUSSION

The ZHB was initiated to provide recommended age-appropriate Zika-related pediatric health and developmental screenings for children born to mothers with laboratory evidence of possible ZIKV infection during pregnancy. Thirty-three children in a jurisdiction with reduced access to pediatric healthcare specialists received the recommended Zika-related pediatric health screenings at the 2021 ZHB. New and continuing medical and developmental concerns were identified and appropriate referrals for follow-up care and services were provided. We identified and described differences in the types of referrals made from the 2018 to the 2021 ZHB. This is likely related to the difference in the ages of the children at the time of their evaluation at each brigade, as this cohort is older. Using the heath brigade model, we achieved our goal of connecting participants to services not previously received. There were no known or reported COVID-19 cases during or after the ZHB from participants, family, or staff; thus, the COVID-19 protocols put in place for the ZHB were successful in mitigating COVID-19 spread.

There are limitations that may have affected the results of this health brigade, mainly the limited number of participants that attended and received screenings. Many phone numbers for the USZPIR families we attempted to contact were no longer in service and the families were unreachable. Many of these phone numbers were collected via surveillance in the USVI DOH Zika response beginning in 2016, and contact numbers may have changed. The reasons for the missed appointments (*n* = 10) were not known, but possible explanations are time constraints, scheduling inconsistencies, perceived lack of need, and fear of in-person clinical settings during the pandemic. Increased number of referrals for participants of the 2021 ZHB may be due to participants that attended with a known concern or delay at the time of their appointment. Developmental delays observed in the children during the ZHB may be multi-factorial and relate to ZIKV infection and/or environmental factors, complications during pregnancy, and/or other factors (Centers for Disease Control and Prevention, 2022). Families were connected with local services in the territory such as audiology and therapies, but the limitation remains for pediatric specialty medical providers in the USVI.

Since the 2018 ZHB many of the children have not received follow-up visual or hearing screenings due to lack of access. While attendance for the 2021 ZHB was low, increased access to specialty care and early intervention services remains important for high-risk children and has been shown to be associated with improvements in cognitive and academic performance (Rosenberg, Zhang, & Robinson, 2008). There is still an ongoing need for pediatric specialty medical providers in the USVI, this is being addressed by the department of health and the government. Routine access to affordable, yearly screenings with improved coordination of care across specialties through a repeated health brigade model could improve care for infants and children in the USVI, while reducing barriers for families. Continued coordination of efforts between public health and clinical practitioners may help further build capacity for sustainable, routine care, and ensure coordination of services for affected children and their families, especially in areas with limited access to specialty care.

## Figures and Tables

**TABLE 1:** Selected demographic characteristics among 33 U.S. Virgin Islands 2021 Zika health brigade participants tracked by U.S. Zika Pregnancy and Infant Registry

Characteristic		*N*	%
Health brigade site	St. Thomas	19	58%
	St. Croix	14	42%
Sex	Female	15	45%
	Male	18	55%
Ages (months)	36–47	10	30%
	48–59	22	67%
	60–71	1	3%
2018 health brigade participant	Yes	24	73%
	No	9	27%

**TABLE 2:** Referrals for specialty care follow-up (*n*, %) among 33 U.S. Virgin Islands 2021 Zika health brigade participants (median age = 49 months) and 88 U.S. Virgin Islands 2018 Zika health brigade participants (median age = 8 months) tracked by U.S. Zika Pregnancy and Infant Registry

		2021 health brigade		2018 health brigade	
Characteristic		*N*	%	*N*	%
Participant received referral for follow-up care	Yes	15	45%	62	70%
	No	18	55%	26	30%
Types of referrals or recommendations for specialty follow-up care	Ophthamology	6	18%	11	13%
	Audiology	1	3%	38	43%
	Neurology	5	15%	10	11%
	Developmental	2	6%	11	13%
	Speech therapy	10	30%	8	9%
	Physical therapy	4	12%	13	15%
	Neuroimaging	1	3%	1	1%
	Ear nose and throat	0	0%	2	2%
	Infants and toddlers (a)	-	-	25	28%
	Head start (b)	1	3%	-	-
ASQ-3 (c) category with a borderline (d) or below cut-off-score (e) and needing further assessment	Communication	11	33%	13	15%
	Gross motor	2	6%	11	13%
	Fine motor	9	27%	16	18%
	Problem solving	7	21%	7	8%
	Personal social	5	15%	8	9%

*Note*: (a) The age of inclusion for the Infants and Toddlers Program is birth through two years, (b) Head Start serves children aged three and four years. (c) ASQ-3 denotes the Ages and Stages Questionnaire, third edition developmental screening. (d) A borderline ASQ-3 score is defined as a score that is between 1 and 2 standard deviations below children's mean performance in each developmental area and behaviors of concern should be reviewed and monitored (Ages and Stages, 2021). (e) A below-cut-off ASQ-3 score is defined as a score at least two standard deviations below children's mean performance in each developmental area and further assessment with a professional may be needed (Ages and Stages, 2021).

## Data Availability

The data that support the findings of this study are available on request from the corresponding author. The data are not publicly available due to privacy or ethical restrictions.
